# Complications And Controversies Of Regional Anaesthesia: A Review

**Published:** 2009-10

**Authors:** Anil Agarwal, Kamal Kishore

**Affiliations:** 1 Professor, Department of Anaesthesiology, Sanjay Gandhi Post Graduate Institute of Medical Sciences, Lucknow 226 014, INDIA; 2 Assistant Professor Anaesthesiology, Department of Anaesthesiology, Sanjay Gandhi Post Graduate Institute of Medical Sciences, Lucknow 226 014, INDIA

**Keywords:** Regional anaesthesia, Complications, Controversies

## Abstract

**Summary:**

Complications of regional anaesthesia has been recognised from very long time. Fortunately serious complication are rare. Safe, effective practice of neuraxial anaesthesia requires a detailed knowledge of potential complications, their incidence and risk factors associated with their occurrence. The incidence of complication were higher for spinal than for epidural anaesthesia. These complications being rare, so existing studies are mainly retrospective, providing information about incidence and their associations but not necessarily demonstrate causality.

There are many areas of controversies regarding the usage of regional anaesthesia i.e. in outpatient surgical procedures, epidural test dose, its safety in infected / febrile / immuno compromised patients, / in patients with neurological disorder and in patients receiving anti-coagulants. Recommendations proposed may be acceptable based on the judgment of the responsible anaesthesiologist. The consensus statements are designed to encourage safe and quality patient care but cannot guarantee a specific outcome.

## Complications of Regional Anaesthesia

Complications of regional anaesthesia have been recognised since Bier reported the first spinal anaesthetic over 100 year ago.^1^ Fortunately, serious complications of neuraxial anaesthesia remain rare but can be devastating when they occur. Because of their rarities, definitive studies of complications remain problematic. Thus, most of the existing studies are retrospective surveys to provide valuable information about incidence and their possible associations.

**Incidence:** of neurologic central neuraxial blockade (CNB) complications is estimated to be between 1/1000 and 1/1,000,000.^2^–^5^ A very large survey of regional anaesthesia from France showed relatively low incidence of serious complications of regional anaesthesia^6^. The incidence of complications was higher for spinal than for epidural anaesthesia.The majority of instances of fatal cardiac arrest could not be directly attributed to spinal anaesthesia. Eighty five percent of patients with neurological deficits had complete recovery within three months.^6^ These complications may be caused either due to mechanical injury from needle or catheter placement and /or adverse physiological responses and /or drug toxicity.

## Individual complications of regional anaesthesia:

**1. Post dural puncture headache:**Bier while describing the first spinal anaesthetic also provided the first description of post dural puncture headache (PDPH)^1^. PDPH is one of the most common complication of neuraxial block, with an overall incidence that may be as high as 7%.^7^ Any breach in the dura mater, which may follow a spinal anaesthetic, an epidural “wet tap”, diagnostic lumber puncture, or migration of epidural catheter may result in PDPH. The mechanism of PDPH is thought to be persistent leakage of cerebrospinal fluid (CSF) through the dural defect at a rate faster than that of CSF production. The transdural leak leads to decreased CSF volume and pressure. During upright position, gravity causes traction on highly innervated meninges and pain sensitive intracranial vessels, which refer pain to the frontal, occipital and neck and shoulder region via trigeminal, glossopharyngeal and vagus and upper cranial nerves respectively.^8^ The diagnosis is basically clinical, usually presents 48-72 hrs after the procedure, typically bilateral, fronto – occipital extending up to neck and shoulders. Pain is described as dull or throbbing; usually associated with nuchal stiffness and backache. The hallmark of PDPH is that it is postural in nature. It often subsides during supine position and may be associated with malaise, photophobia, nausea, vomiting and cranial nerve palsies.

Subdural hematoma is rare but is most severe complication of PDPH.^9^ The risk factors of PDPH are young age, female sex, pregnancy and prior history of PDPH.^10^ Use of smaller and non cutting (Whitacre) needles decreases the incidence of PDPH.^11^

As far as treatment is concerned, it could be conservative or invasive. The conservative measures include bed rest, hydration, analgesics, abdominal binders and caffeine. These measures will decrease downward traction, increase CSF production, constrict the intracranial vessels and provide the symptomatic relief.^12^

The invasive treatment is epidural blood patch, which is considered to be most effective treatment in complete resolution of most of the symptoms^13^.Aseptically withdrawn autologous blood is injected in the same space or one space below until the patient experiences lumber discomfort or until 20 ml has entered in epidural space.

**2. Backache:** Backache is a frequent complaint of neuraxial anaesthesia. Although incidence is high but neuraxial anaesthesia may not be the sole cause.^14^ The frequency of backache is approximately similar after spinal or general anaesthesia.^15^ Localised trauma to the intervertebral disk or excessive stretching of associated ligaments after loss of lumber lordosis due to relaxation of paraspinal muscles are supposed to be the causative factors. The pain is usually mild and self limiting although it may last for several weeks. Nonsteroidal anti-inflammatory agents and warm or cold compresses are sufficient for backache. Although backache is usually benign, it may be an indication of more serious complications like epidural abscess, spinal hematoma or syndrome of transient neurologic symptoms.

**3. Transient Neurological symptoms:** Transient neurological symptoms (TNS) were first reported in 1993 by Schneider et al who described the development of severe radicular back pain after resolution of an uneventful, lidocain spinal anaesthetic.^16^ There was no sensory or motor deficit and no signs of bowel and bladder dysfunction. The symptoms resolved within one week. The aetiology of TNS is not well defined. However, up to 30% of patients with TNS report severe pain.^17^ Zoric et al in their systemic review analysed that the use of lidocaine for spinal anaesthesia increased the risk of developing TNS. There was no evidence that this painful condition was associated with any neurologic pathology; the symptoms disappeared spontaneously by the fifth postoperative day. The risk of developing TNS after spinal anaesthesia with lidocain was significantly higher than when bupivacaine, prilocaine, or procaine were used.^18^ Freedman's study identified other risk factors for the development of TNS besides lignocain: outpatient status, obesity and lithotomy position.^17^

**4. Total spinal anaesthesia:** Total spinal anaesthesia can happen when there is unintentional intrathecal administration of local anaesthetics during epidural or caudal anaesthesia. The onset is usually rapid. Patient exhibits signs of cardiovascular collapse in the form of severe hypotension, bradycardia and respiratory insufficiency. Careful aspiration, use of test dose and incremental drug dosing can help avoid this complication. If total spinal anaesthesia occurs, then patients are put in trandlenburg position so as to increase venous return, administer fluid along with inotropic support to raise blood pressure, may need tracheal intubation to support ventilation. Fortunately need for sedation during intubation and mechanical ventilation is minimal. At very high dose pupil may also dilate which come to normal size as the effect of local anaesthetic recedes.

**5. Spinal or epidural hematoma:** Epidural or spinal haematoma is a rare, but potentially disastrous complication of central neuraxial blocks. It was initially reported within 10 years after administration of first spinal anaesthetic.^19^ The incidence of such hematomas has been estimated to be about 1:150,000 for epidural blocks and 1:220,000 for spinal anaesthetics.^20^ Spinal hematoma is particularly catastrophic as it may go unnoticed until there is permanent neurologic compromise. The variables associated with increased incidence spinal hematoma are; female gender, increased age, traumatic needle /catheter placement, indwelling epidural catheter placement during, immediate preoperative, intra-operative and postoperative LMWH administration.^21^ Patient usually present with sudden new onset sharp back and leg pain with numbness, weakness, bladder and bowel dysfunction. When spinal hematoma is suspected, neurologic imaging (MRI and CT scan) and neurologic consultation should be immediately obtained. Good neurological recovery is seen in patients who have undergone surgical decompression within 8-12 hours.

**6. Epidural abscess:** Epidural abscess is a serious complication after neuraxial block. The incidence varies from 0.015% to 0.7% according to different studies.^22^,^23^ Although epidural abscess is uncommon, early diagnosis and treatment is paramount. Symptoms of epidural abscess usually begin several days after neural block, sometimes after months, include back pain, fever, malaise, signs of cord compression including sensory changes, flaccid paralysis followed by spastic paralysis, elevated blood leukocytes count, elevated cerebrospinal fluid protein and leukocytes.^22^,^24^ Clinicalsigns, duration of symptoms and the rate of neurological deterioration show a high inter-individual variability, and the classic triad(spinal pain, fever and neurological deficit) is often not found, especially not at first presentation to a physician.^25^

Gadolinium-enhanced magnetic resonance imaging is the most sensitive, specific and accurate imaging method.^25^–^27^ Staphylococci are the most frequent etiologic agents (57%) followed by streptococci (18%) and gram negative bacilli (13%).^22^ Associated risk factors are diabetes mellitus, chronic renal failure, epidural or systemic steroid injection, herpes zoster and chronic alcohol abuse.

The management of epidural abscess involve, drainage of the abscess and eradication of the microorganism as the basic principles of therapy. Surgical therapy is the treatment of choice in the overwhelming majority of cases. Rapid surgical intervention is not only needed to minimize neurological damage, but also for controlling sepsis.Duration of antimicrobial treatment is usually 4–6 weeks for epidural abscess.^28^

**7. Meningitis:** Dural puncture may be a risk for infection of subarachnoid space. Exact mechanism by which bacteria reaches to the central nervous system may not be known but the infectious source may be exogenous (e.g., contaminated equipment or medication) or endogenous (a bacterial source in the patient seeding to the needle or catheter site). Microorganisms can also be transmitted via a break in aseptic technique, and indwelling catheters may be colonized from a superficial site (skin) and subsequently serve as a wick for spread of infection from the skin to the epidural or intrathecal space. The symptoms appear hours to days after anaesthesia, sometimes onset time may be up to one month.^29^ Initial clinical presentation are fever and headache, with backache with emesis, classical sign of meningism and lithargy. CSF is usuallyturbid with raised leukocytes, proteins and low glucose concentration. In great majority of cases the causative organism is alpha-haemolytic streptococcus.^30^ Lumbar puncture aids diagnosis. Give appropriate antibiotics early, which will usually be before the causative or its sensitivity is established. Use of steroid is debatable but recommended for community acquired meningitis.^31^

**8. Arachnoiditis:** Arachnoiditis, another rare complication of neuraxial anaesthesia may appear as transient nerve root irritation, cauda equina, and conus medullaris syndromes. It may show its presence later as radiculitis, clumped nerve roots, fibrosis, scarring dural sac deformities, pachymeningitis, pseudomeningocele, and syringomyelia, etc., all associated with arachnoiditis. Regarding regional anaesthesia in the neuraxis, arachnoiditis has resulted from epidural abscesses, traumatic punctures (blood), local anaesthetics, detergents, antiseptics or other substances unintentionally injected into the spinal canal. Patients usually presents with pain in the lower back, dysesthesia and numbness not following the usual dermatome distribution.^32^

**9. Cardiac Arrest:** The incidence and causes of cardiac arrest related to anaesthesia in the perioperative period have been studied over two decades in many countries.^33^–^35^ Majority of the literature regarding cardiac arrest related to regional anaesthesia involves retrospective studies or case reports. Few prospective surveys assessing a large number of patients have been published.^36^ The incidence of cardiac arrest during regional anaesthesia varies in different studies and it ranges from 1.5-6.4/10000 cases.^37^–^38^ Recently Charuluxananan et al reported the incidence of cardiac arrest following spinal anaesthesia is 2.73/10000 patients.^39^ Theories regarding the mechanism by which neuraxial block contributes to cardiac arrest involve a circulatory aetiology. Initially sedation was speculated to have contributed to many of the cardiac arrests during spinal anaesthesia.^40^ Another likely cause could be decrease in preload associated with neuraxial block resulting in a shift in cardiac autonomic balance toward the parasympathetic system leading to bradycardia. At least three mechanisms have been proposed, including activation of the low-pressure baroreceptors in the right atrium, the receptors within the myocardial pacemaker cells, and mechanoreceptors in the left ventricle (stimulating a paradoxical Bezold-Jarisch response). In addition, a high sympathetic level may directly favour vagal tone; sedation, hypoxemia, hypercarbia, and chronic medications (such as [beta]-adrenergic antagonists) may contribute to the development and severity of bradycardia.^41^ Intravascular fluid administration, the administration of mixed [alpha]- and [beta]-agonists, and vagolytic therapy have all been advocated to decrease the frequency of and improve the survival associated with cardiac arrest during neuraxial block. 42

**10. Urinary retention:** Neuraxial anaesthesia blocking S2-S4 nerve root fibres decreases the urinary bladder tone and inhibits the voiding reflex. Urinary retention is common after anaesthesia and surgery, reported incidence of between 5% and 70%. Co morbidities, type of surgery, and type of anaesthesia influence the development of postoperative urinary retention (POUR).^43^ Lower concentrations of local anaesthetic are needed for paralysis of urinary bladder than motor nerves of lower extremities. Ultrasound has been shown to provide an accurate assessment of urinary bladder volume and a guide to the management of POUR. Recommendations for urinary catheterization in the perioperative setting vary widely, influenced by many factors, including surgical factors, type of anaesthesia, co morbidities, local policies, and personal preferences. Inappropriate management of POUR may be responsible for bladder over distension, urinary tract infection, and catheter-related complications.

**11. Drug Toxicity:** Epidural anaesthesia can potentially produce local anaesthetic drug toxicity via intravascular administration of drug in epidural vein.All local anaesthetic agents block neuronal voltage-gated sodium channels, and thus suppress conduction in peripheral nerves. Systemic accumulation of local anaesthetic agents may affect the functional integrity of these structures.All local anaesthetics can cause CNS toxicity and cardiovascular toxicity if their plasma concentrations are increased by accidental intravenous injection, with the CNS affected at lower blood levels. With regard to CNS toxicity the plasma levels necessary to provoke CNS symptoms are to a large extent agent-specific. Initially, these toxic mechanisms are due to a selective blockade of cortical inhibitory neurons, which enables the formation of seizure potentials within sub cortical structures. Excitation of the CNS may be manifested by numbness of the tongue and perioral area, and restlessness, which may progress to seizures, respiratory failure and coma. Treatment of CNS toxicity includes maintaining adequate ventilation and oxygenation, and controlling seizures with the administration of thiopental sodium or benzodiazepines. Cardiovascular toxicity generally begins after signs of CNS toxicity have occurred. Bupivacaine and etidocaine appear to be more cardio toxic than most other commonly used local anaesthetics. Direct cardiac effects of local anaesthetics can be divided into (i) stereo specific inhibition of intracardial conduction and (ii) unspecific inhibition of myocardial energy supply and ion channels. The corresponding spectrum of symptoms is not uniform and may range from extreme bradycardia, (malignant) ventricular arrhythmia to refractory cardiac arrest.^44^ Treatment of cardiovascular toxicity depends on the severity of effects. Cardiac arrest caused by local anaesthetics should be treated with cardiopulmonary resuscitation procedures, but bupivacaineinduced dysrhythmias may be refractory to treatment. Injection of local anaesthetic through micro catheters and possibly small-gauge spinal needles results in poor CSF mixing and accumulation of high concentrations of local anaesthetic in the areas of the lumbosacral nerve roots. In contrast to bupivacaine, the hyperbaric lidocain (lignocain) formulation carries a substantial risk of neurotoxicity when given intrathecally. Reduction of hepatic blood flow by drugs or hypotension will decrease the hepatic clearance of amide local anaesthetics. Special caution must be exercised in patients taking digoxin, calcium antagonists and/or beta-blockers.^45^

## Controversies Regarding Regional Anaesthesia:

Despite using the neuraxial block for so many years, the controversies related to the appropriate use of these blocks still remains are as follows:

**1. Regional anaesthesia for outpatients:** Earlier it was believed that regional anaesthesia was not fit for outpatient anaesthesia; however, an ever increasing number of day-case surgical patients are putting up a challenge on the existing methods of anaesthesia for day care procedures. ‘Walk-in, walk-out’ spinals with an extremely low dose of lidocaine and opioids for gynaecological laparoscopy created the concept of selective spinal anaesthesia. Reintroduction of chloroprocaine may provide a solution for bilateral, shortacting spinal anaesthesia in the future. To produce reliable spinal anaesthesia with a reasonable recovery time it is essential to understand the factors affecting the spread of spinal block and to choose the optimal drug and adequate dose for specific surgical procedures.^46^

**2. Epidural test dose:** In epidural anaesthesia a large volume of local anaesthetic is used, which if injected intrathecally or intravascularly, can cause significant toxicity. So the classical epidural test dose combining 3 ml of 1.5% lidocaine with 1:200,000 epinephrine is used. Most controversy surrounds the use of test dose in obstetric patients, in whom blood flow to the uterus may be decreased by intravascular injection, thereby jeopardizing the foetus.^47^ Epidural test dose may not be reliable in patients on β-blockers or in patients undergoing epidural catheter placement under general anaesthesia. At times aspiration may produce false negative results. Recently Mhyre et al, evaluated the different strategies proposed to minimize the incidence of epidural vein cannulation during lumbar epidural catheter placement in pregnant women, and concluded that this risk could be reduced with the lateral patient position, fluid pre-distension of the epidural space, a single orifice catheter, a wire-embedded poly-urethane epidural catheter and limiting the depth of catheter insertion to < 6 cm.^48^ Norris et al proposed that there was no justification in administering test dose in patients where aspiration was negative.^49^ Still many clinicians follow the golden rule of initial aspiration and incremental doses of local anaesthetics.

**3. Use of neuraxial block in infected or febrile patients:** Use of single shot spinal or short term epidural anaesthesia poses little risk to the patient who may become transiently bacteramic during surgery. Conversely neuraxial blocks in infected or febrile patients may increase the risk of neuraxial infection, and these blocks remain controversial. Most authorities believe that neuraxial blocks should not be performed in patients who are bacteramic. Conservatively, all patients with an established local or systemic infection should be considered at risk for developing infection of the CNS. Available data suggest that:

Serious central neuraxial infections such as arachnoiditis, meningitis, and abscess after spinal or epidural anaesthesia are rare. The decision to perform a regional anaesthetic technique must be made on an individual basis considering the anaesthetic alternatives, the benefits of regional anaesthesia, and the risk of CNS infection (which may theoretically occur in any bacteramic patient). Despite conflicting results, many experts suggest that, except in the most extraordinary circumstances, central neuronal block should not be performed in patients with untreated systemic infection. Available data suggest that patients with evidence of systemic infection may safely undergo spinal anaesthesia, provided appropriate antibiotic therapy is initiated before dural puncture and the patient has shown a response to therapy, such as a decrease in fever (placement of an indwelling epidural (or intrathecal) catheter in this group of patients remains controversial). A delay in diagnosis and treatment of major CNS infections of even a few hours may significantly worsen neurologic outcome.^50^

**4. Neuraxial anaesthesia for immuno com-promised patients:** Neuraxial anaesthesia and analgesia are advantageous over systemic opioids in providing better analgesia, reduced pulmonary complications and reduced graft occlusion. In addition, neuraxial analgesia may decrease the risk of infection through attenuation of the stress response and preservation of immune function.^51^ Despite these benefits, patients with altered immune status because of neoplasm, immuno-suppression after solid organ transplantation, and chronic infection with human immunodeficiency virus (HIV) or herpes simplex virus (HSV) are often not considered candidates for neuraxial techniques because of the risk of infection around the spinal cord or within the spinal canal. A depressed immune state increases both frequency and severity of infection. The relative risk of central nervous system (CNS) infections in patients with altered immune status compared with the normal host is unknown but still Horlocker et al suggests that the decision to perform a regional anaesthetic technique must be made on an individual basis considering the anaesthetic alternatives, the benefits of regional anaesthesia, and the risk of CNS infection (which theoretically are more likely to occur in the immuno compromised patient), as well as the risk of hemorrhagic or neurologic complications.^52^

The attenuated inflammatory response within the immuno compromised patient may diminish the clinical signs and symptoms often associated with infection. Likewise, the range of microorganisms causing invasive infection in the immuno compromised host is higher than that affecting the general population and includes atypical and opportunistic pathogens. Consultation with an infectious disease specialist is advised to facilitate initiation of early and effective therapy.

A delay in the diagnosis and treatment of CNS infections worsens neurologic outcome and increases mortality. There are inadequate data available regarding the safety of spinal and epidural anaesthesia in the presence of primary Herpes simplex virus-2 infection. However, viremia, fever, and meningitis have been reported. These findings would suggest a conservative approach. Central neuronal block has been shown to be safe in patients with recurrent HSV infections, although exacerbations of HSV-1 have been reported in association with intrathecal and epidural opioids. Minimal data suggest that neuraxial and peripheral techniques (including epidural blood patch) can be performed safely in HIV-infected patients. The presence of preexisting neurologic pathology is common in these patients and must be considered^52^

**5. Neuraxial blocks in neurological disorder.** Historically, the use of regional anaesthetic techniques in patients with pre-existing central nervous system (CNS) disorders has been considered relatively contraindicated. The fear of worsening neurologic outcome secondary to mechanical trauma, local anaesthetic toxicity, or neural ischemia is commonly reported. Many clinicians completely avoid neuraxial block in patients with pre-existing neurologic disorder because of medico legal implications of any increase in postoperative neurologic deficit. Many earlier studies proposed that there is exacerbation of neurological deficit after regional anaesthesia, Horlocker^53^ et al conducted a retrospective review and analysed that the decision to perform regional anaesthesia in patients with pre-existing neurologic deficits should be based on the risks and potential benefits of each individual case. He concluded that the risks commonly associated with neuraxial anaesthesia and analgesia in patients with pre-existing CNS disorders may not be as frequent as once thought and that neuraxial blockade should not be considered an absolute contraindication within this patient population.

**6. Neuraxial blocks in diabetic patients:** Peripheral sensory motor neuropathies may occur secondary to a variety of underlying aetiologies, including metabolic, autoimmune, infectious, or hereditary abnormalities. Of these aetiologies, diabetes mellitus is the most common cause of systemic polyneuropathy. The frequency of diabetic polyneuropathy ranges from 4% to 8% at the time of initial presentation, to approximately 50% in patients with chronic disease. Ultimately, all asymptomatic patients will likely be found to have abnormalities of nerve conduction.^54^ Patients with underlying, chronic neural compromise secondary to is-chemic (peripheral vascular disease or microangiopathy), toxic (chemotherapy), or metabolic (diabetes mellitus) abnormalities may be at an increased risk of further neurologic injury because of a physiologic “double-crush.” The pathophysiology of diabetic poly-neuropathy is multifactorial and not completely understood. Any disruption in the supply of essential components (blood, oxygen, adenosine triphosphate, glucose) to the axon can cause distal axonal degeneration. There are several types of neuropathies associated with diabetes, each classified into a distinct clinical syndrome. Distal symmetric sensorimotor polyneuropathy is the most common syndrome, and is often considered synonymous with the term diabetic neuropathy. It has been suggested that when compared with the general population, patients with pre-existing neural compromise, including peripheral sensorimotor neuropathy and diabetic polyneuropathy, may be at an increased risk of perioperative nerve damage after regional blockade.^55^ Abnormal local anaesthetic diffusion and subsequent neurotoxicity may have been the contributing factors to the neurological complications. Toxicity differs greatly among local anaesthetics, bupivacaine being the least toxic. Even though hyperbaric lidocaine is most often associated with neurotoxicity, all local anaesthetics are potentially neurotoxic.^56^ Using rats injected with streptozotocin to induce diabetes, Kalichman et al concluded that local anaesthetic requirements are decreased in diabetic animals, and therefore the risk of local anaesthetic toxicity and subsequent nerve injury is increased.^57^ Finally, it is possible that epinephrine may have a pathogenic role in the development of neurotoxicity after regional anaesthesia. Epinephrine alone, or when combined with a local anaesthetic, may significantly reduce nerve blood flow. Although there is no human study to confirm the decreased requirement of local anaesthetics in neuraxial blocks in diabetic neuropathy patients; however, the risk appears to be higher than that reported for the general population. Clinicians should be aware of this potentially high-risk subgroup of patients when developing and implementing a regional anaesthetic care plan.^58^

**7. Premedication with benzodiazepines:** There is widely held belief that benzodiazepines should be used with neuraxial anaesthesia to decrease the systemic toxicity by elevating seizure threshold. But data indicates that resuscitation may become more difficult after cardiovascular collapse after bupivacaine if diazepam is used as premedication.

**8. Neuraxial blocks in patients receiving anticoagulants:** Numerous studies have documented the safety of neuraxial anaesthesia and analgesia in the anticoagulated patient. Patient management is based on appropriate timing of needle placement and catheter removal relative to the timing of anticoagulant drug administration.

The rarity of spinal hematoma defies a prospective-randomized study, and there is no current laboratory model. As a result, these consensus statements represent the collective experience of recognized experts in the field of neuraxial anaesthesia and antico agulation. They are based on case reports, clinical series, pharmacology, haematology, and risk factors for surgical bleeding. The decision to perform spinal or epidural anaesthesia/analgesia and the timing of catheter removal in a patient receiving antithrombotic therapy should be made on an individual basis, weighing the small, though definite risk of spinal hematoma with the benefits of regional anaesthesia for a specific patient. Vigilance in monitoring is critical to allow early evaluation of neurologic dysfunction and prompt intervention. We must focus not only on the prevention of spinal hematoma, but also optimization of neurologic outcome.^59^

Neuraxial anaesthetic techniques remain an important part of anaesthesiologist's armamentarium. Depending on specific patient factors and the setting in which these techniques are applied, they may offer significant advantages. The occurrence of any complication implies an interaction of factors related to the blockade itself and known or unknown pre-existing conditions in the patient. Fortunately, neuraxial techniques are associated with a low incidence of significant complications. However, as over all perioperative safety improves, rare and potentially devastating complications from neuraxial anaesthesia become more significant. Anaesthesiologists can minimise the risk of these complications by appropriately applying neuraxial techniques in carefully selected patients. Proper patient selection depends on detailed knowledge of any conditions that may predispose patients to complications from neuraxial techniques. Regarding controversies in regional anaesthesia variances from recommendations contained here may be acceptable based on the judgment of the responsible anaesthesiologist. The consensus statements are designed to encourage safe and quality patient care but cannot guarantee a specific outcome. They are also subject to timely revision as justified by evolution of information and practice. Finally, the investigation does suggest that the risks commonly associated with neuraxial anaesthesia in patients with preexisting CNS disorders, immunocompromised patients, patients with infection, patients on anticoagulants and diabetic patients, may not be as frequent as once thought. In fact, it may be prudent to reconsider the long-standing belief that neuraxial anaesthesia be considered an absolute contraindication within this patient population. However, to make definitive conclusions on the safety of these techniques in these patients require further prospective study.

**Table:** Recommended guidelines for performing spinal procedures in anticoagulated patients

Warfarin	Discontinue chronic warfarin therapy 4–5 days before spinal procedure and evaluate INR. INR should be with in the normal range at time of procedure to ensure adequate levels of all vitamin K-dependent factors.
Antiplatelet medications	No contraindications with aspirin or NSAIDs. Thienopyridine derivatives (clopidogrel and ticlopidine) should be discontinued 7 days and 14 days, respectively, prior to procedure. GP IIb/IIIa inhibitors should be discontinued to allow recovery of platelet function prior to procedure (8 hours for tirofiban and eptifibatide, 24-48 hours for abciximab).
Thrombolytics/fibrinolytics	There are no available data to suggest a safe interval between procedure and initiation or discontinuation of these medications. Follow fibrinogen level and observe for signs of neural compression.
LMWH	Delay procedure at least 12 hours from the last dose of thromboprophylaxis LMWH dose. For "treatment" dosing of LMWH, at least 24 hours should elapse prior to procedure. LMWH should not be administered within 24 hours after the procedure.
Unfractionated SQ heparin	There are no contraindications to neuraxial procedure if total daily dose is less than 10,000 units. For higher dosing regimens, manage according to intravenous heparin guidelines.
Unfractionated IV heparin	Delay spinal puncture 2–4 hours after last dose, document normal aPTT. Heparin may be restarted 1 hour following procedure.

**Note:**—NSAIDs indicates nonsteroidal anti-inflammatory drugs; GP IIb/IIIa, platelet glycoprotein receptor IIb/IIIa inhibitors; INR, international normalized ratio; LMWH, low-molecular-weight heparin; aPTT, activated partial thromboplastin time.

## References

[1] Bier A (1899). Versuche ueber cocainisirung des rueckemarkes Deutche Zeitschrift fuer chirurgie.

[2] Aromaa U, Lahdensuu M, Cozanitis DA (1997). Severe complications associated with epidural and spinal anaesthesias in Finland 1987–1993. A study based on patient insurance claims. Acta Anaesthesiol Scand.

[3] Chiapparini L, Sghirlanzoni A, Pareyson D, Savoiardo M (2000). Imaging and outcome in severe complications of lumbar epidural anaesthesia: report of 16 cases. Neuroradiology.

[4] Dahlgren N, Tornebrandt K (1995). Neurological complications after anaesthesia. A follow-up of 18,000 spinal and epidural anaesthetics performed over three years. Acta Anaesthesiol Scand.

[5] Horlocker TT, Heit JA (1997). Low molecular weight heparin: biochemistry, pharmacology, perioperative prophylaxis regimens, and guidelines for regional anesthetic management. Anesth Analg.

[6] Auroy Y, Narchi P, Messiah A (1997). Serious complications related to regional anesthesia: results of a prospective survey in France. Anesthesiology.

[7] Halpern S, Preston R (1994). Postdural puncture headache and spinal needle design. Anesthesiology.

[8] Brownridge P (1983). The management of headache following accidental dural puncture in obstetric patients. Anaesthesia Intensive Care.

[9] Duarte WL, Araújo Fde S, Almeida MF, Geber DG, de Castro CH (2008). Subdural hematoma after inadvertent dura mater puncture. Case report. Rev Bras Anestesiol.

[10] Rasmussen BS, Blom L, Hansen P (1989). Postspinal headache in young and elderly patients. Two randomised double blind studies that compare 20- and 25- gauze needles. Anaesthesia.

[11] Arendt K, Demaerschalk BM, Wingerchuk DM, Camann W (2009). Atraumatic lumbar puncture needles: after all these years, are we still missing the point?. Neurologist.

[12] Turnbull DK, Shepherd DB (2003). Post-dural puncture head-ache: pathogenesis, prevention and treatment. Br J Anaesth.

[13] van Kooten F, Oedit R, Bakker SL, Dippel DW (2008). Epidural blood patch in post dural puncture headache: a randomised, observer-blind, controlled clinical trial. J Neurol Neurosurg Psychiatry.

[14] Kely D, Hewitt SR (1972). Lumbar epidural block in labour: A clinical analysis. British journal Anaesthesia.

[15] Brown EM, Elman DS (1961). Postoperative backache. Anesthesia Analgesia.

[16] Schneider M, Ettlin T, Kaufmann M (1993). Transient neurologic toxicity after hyperbaric subarachnoid anaesthesia with 5% lidocain Anesthesia Analgesia.

[17] Freedman JM, Li DK, Drasner K (1998). Transient neurologic symptoms after spinal anaesthesia: An epidemiologic study of 1863 patients. Anesthesiology.

[18] Zaric D, Pace NL (2009). Transiet neurologic symptoms (TNS) following spinal anaesthesia with lidocaine versus other local anaesthetics. Cochrane Database Syst Rev.

[19] Horlocker TT, Wedel DJ (1998). Anticoagulation and neuraxial block: historical perspective, anesthetic implications, and risk management. Reg Anesth Pain Med.

[20] Tryba M (1993). Epidural regional anesthesia and low molecular heparin. Anasthesiol Intensivmed Notfallmed Schmerzther.

[21] Horlocker TT, Wedel DJ, Benzon H, Brown DL, Enneking FK, Heit JA, Mulroy MF (2003). Rosenquist RW, Rowlingson J, Tryba M, Yuan CS. Regional anesthesia in the anticoagulated patient: defining the risks (the second ASRA Consensus Conference on Neuraxial Anesthesia and Anticoagulation). Reg Anesth Pain Med.

[22] Kindler CH, Seeberger MD, Staender SE (1998). Epidural abscess complicating epidural anesthesia and analgesia. An analysis of the literature. Acta Anaesthesiol Scand.

[23] Holt HM, Andersen SS, Andersen O, Gahrn-Hansen B, Siboni K (1995). Infections following epidural catheterization. J Hosp Infect.

[24] Wedel DJ, Horlocker TT (1996). Risks of regional anaesthesiainfectious, septic. Regional Anesth.

[25] Sendi P, Bregenzer T, Zimmerli W (2008). Spinal epidural abscess in clinical practice QJM.

[26] Heusner AP (1948). Nontuberculous spinal epidural infections. N Engl J Med.

[27] Patel D, Baron EM, Enochs WS, Ruth C, Harrop JS, Vaccaro AR (2008). Spinal epidural abscess mimicking lymphoma: a case report. Orthopedics.

[28] Bluman EM, Palumbo MA, Lucas PR (2004). Spinal epidural abscess in adults. J Am Acad Orthop Surg.

[29] Rodrigo N, Perera KN, Ranwala R, Jayasinghe S, Warnakulasuriya A, Hapuarachchi S (2007). Aspergillus meningitis following spinal anaesthesia for caesarean section in Colombo, Sri Lanka. Int J Obstet Anesth.

[30] Bouhemad B, Dounas M, Mercier FJ, Benhamou D (1998). Bacterial meningitis following combined spinal-epidural analgesia for labour. Anaesthesia.

[31] Baer ET (2006). Post-dural puncture bacterial meningitis. Anesthesiology.

[32] Aldrete JA (2003). Neurologic deficits and arachnoiditis following neuraxial anesthesia. Acta Anaesthesiol Scand.

[33] Morgan CA, Webb RK, Cocklings J, Williamson JA (1993). The Australian Incident Monitoring Study Cardiac arrest: an analysis of 2000 incident reports. Anaesth Intensive Care.

[34] Kawashima Y, Takahashi S, Suzuki M (2003). Anaesthesia-related mortality and morbidity over a 5-year period in 2,363,038 patients in Japan. Acta Anaesthesiol Scand.

[35] Caplan R, Ward R, Posner K, Cheney F (1988). Unexpected cardiac arrest during spinal anesthesia: a closed claims analysis of predisposing factors. Anesthesiology.

[36] Charuluxananan S, Suraseranivongse S, Punjasawadwong Y (2005). The Thai Anesthesia Incidents Study (THAI Study) of anesthetic outcomes: I. Description of methods and populations. J Med Assoc Thai.

[37] Sprung J, Warner ME, Contreras MG (2003). Predictors of survival following cardiac arrest in patients undergoing noncardiac surgery: a study of 518,294 patients at a tertiary referral center. Anesthesiology.

[38] Auroy Y, Narchi P, Messiah A, Litt L, Rowier B, Samii K (1997). Serious complications related to regional anesthesia. Results of a prospective survey in France. Anesthesiology.

[39] Charuluxananan S, Thienthong S, Rungreungvanich M (2008). Cardiac arrest after spinal anesthesia in Thailand: a prospective multicenter registry of 40,271 anesthetics. Anesth Analg.

[40] Geffin B, Sharpiro L (1998). Sinus bradycardia and asystole during spinal and epidural anesthesia: a report of 13 cases. J Clin Anesth.

[41] Liguori GA, Sharrock NE (1997). Asystole and severe bradycardia during epidural anesthesia in orthopedic patients. Anesthesiology.

[42] Kopp SL, Horlocker TT, Warner ME, Hebl JR, Vachon CA, Schroeder DR, Gould AB Sprung (2005). Cardiac arrest during neuraxial anesthesia: frequency and predisposing factors associated with survival. Anesth Analg.

[43] Baldini G, Bagry H, Aprikian A, Carli F (2009). Postoperative urinary retention: anesthetic and perioperative considerations. Anesthesiology.

[44] Zink W, Graf BM (2003). Toxicology of local anesthetics. Clinical, therapeutic and pathological mechanisms. Anaesthesist.

[45] Naquib M, Maqboul MM, Samarkandi AH, Attia M (1998). Adverse effects and drug interactions associated with local and regional anaesthesia. Drug Saf.

[46] Korhonen AM (2006). Use of spinal anaesthesia in day surgery. Curr Opin Anaesthesiol.

[47] Chadwick HS, Benedetti C, Ready LB, Williams V (1987). Epinephrine-containing test doses—don't throw the baby out with the bath water. Anesthesiology.

[48] Mhyre JM, Greenfield ML, Tsen LCPolley LS (2009). A systematic review of randomized controlled trials that evaluate strategies to avoid epidural vein cannulation during obstetric epidural catheter placement. Anesth Analg.

[49] Norris MC, Ferrenbach D, Dalman H, Fogel ST, Borrenpohl S, Hoppe W, Riley A (1999). Does epinephrine improve the diagnostic accuracy of aspiration during labor epidural analgesia?. Anesth Analg.

[50] Wedel DJ, Horlocker TT (2006). Regional anesthesia in the febrile or infected patient. Reg Anesth Pain Med.

[51] Liu S, Carpenter RL, Neal JM (1995). Epidural anesthesia and analgesia. Their role in postoperative outcome. Anesthesiology.

[52] Horlocker TT, Wedel DJ (2006). Regional anesthesia in the immunocompromised patient. RegAnesth Pain Med.

[53] Hebl JR, Horlocker TT, Schroeder DR (2006). Neuraxial anesthesia and analgesia in patients with preexisting central nervous system disorders Anesth Analg.

[54] Ross MA (1993). Neuropathies associated with diabetes. Med Clin North Am.

[55] Horlocker TT, O'Driscoll SW, Dinapoli RP (2000). Recurring brachial plexus neuropathy in a diabetic patient after shoulder surgery and continuous interscalene block. Anesth Analg.

[56] Selander D (1993). Neurotoxicity of local anesthetics: animaldata. Reg Anesth.

[57] Kalichman MW, Calcutt NA (1992). Local anesthetic-induced conduction block and nerve fiber injury in streptozotocin-diabetic rats. Anesthesiology.

[58] Hebl JR, Kopp SL, Schroeder DR, Horlocker TT (2006). Neurologic complications after neuraxial anesthesia or analgesia in patients with preexisting peripheral sensorimotor neuropathy or diabetic polyneuropathy. Anesth Analg.

[59] Horlocker TT, Wedel DJ, Benzon H (2003). Regional anesthesia in the anticoagulated patient: defining the risks (the second ASRA Consensus Conference on Neuraxial Anesthesia and Anticoagulation). Reg Anesth Pain Med.

